# Supplementary material for: Use of Bedinvetmab (Librela®) for Canine Osteoarthritis in France, Germany, Italy, Spain, and the UK: Quantitative Analysis of Veterinarian Satisfaction and Real-World Treatment Patterns
Source: Animals (Basel). 2024 Jul 31;14(15):2231. doi: 10.3390/ani14152231 (PMC11311012; doi:10.3390/ani14152231)
Supplement: Supplementary file 1 [file animals-14-02231-s001.zip › animals-3034787-supplementary.pdf]

Supplementary material

Patient record form example

Instructions for the veterinarian to be displayed in red at the top of the screen:

Considering your practice from February 2021 until now, for the purposes of this study, and to better understand pet owner compliance, please complete the following forms for 5-7 patients meeting the following criteria:

All must have been diagnosed or suspected of Osteoarthritis (OA) pain

<u>All currently being prescribed with bedinvetmab</u>

Patients must be aged 12 months +

Please do not enter any patients who are currently pregnant or lactating animals

In case you have more than 5-7 patients in your practice with the above profile, please choose and describe the most average cases for your practice.

PATIENT DEMOGRAPHICS AND DISEASES STATUS		
D1	Age	_____ years and ___ months
D2	Gender	1. Male<br>2. Female
D3	Weight	____ kg
D4a	Diagnosis	1. Diagnosed OA<br>2. Suspected OA
D4b	Severity stage	1. Mild (early stage) osteoarthritis - dog experiencing intermittent pain and showing intermittent signs of lameness that resolve after rest and that may be more apparent after sudden bouts of exercise)<br>2. Moderate (mid-stage) osteoarthritis - dog experiencing chronic pain and becoming exercise intolerant, may hesitate or show difficulty jumping into car or going up stairs and more likely to lag behind during walks)<br>3. Severe (late stage) osteoarthritis - dog experiencing chronic pain and losing ability to walk and muscle wasting in affected limbs<br><br>**SINGLE CHOICE**

D4c	Do you use a standard method of staging dogs with OA?	1. Yes, please explain: __________<br>2. No
D5a	**If D4a=2:** First suspicion of OA or first recommendation for pain management	|_|_| (MM) |_|_|_|_| (YYYY)
D5b	**If D4a=1**: Date of diagnosis	|_|_| (MM) |_|_|_|_| (YYYY)
D6	Comorbidities or associated conditions	1. Oral infection (Tartar and gingivitis)<br>2. Ear infection<br>3. Itchy skin/ skin infections<br>4. Urinary problems<br>5. Obesity<br>6. Diabetes<br>7. Cardiac disease<br>8. Renal impairment<br>9. Others, please specify: _________
D7a	What were the signs or history that made you suspect OA pain?	1. Agitation during palpation of the limbs and joints<br>2. The dog had difficulty standing/sitting<br>3. The dog was limping or reluctant to walk<br>4. The dog became depressed and/or less social<br>5. Owner reported signs of OA pain and/or change in activity level<br>6. Others, please specify: _____________
D7b	**If D4a=1:**<br>How was the OA diagnosis established?	1. Palpation of the limbs and joints to assess for painful response<br>2. Thickening of the joint capsule<br>3. Accumulation of joint fluid (effusion) or osteophytes<br>4. Muscle atrophy<br>5. X-ray<br>6. Others, please specify: ______<br><br>**MULTIPLE CHOICE**
D8	How many times have you seen this patient to check and follow-up the OA condition / evolution?	Number of years:_____<br><br>Average number of times / year:_________<br><br>**NUMERIC INPUT, ALLOW 1-99**

D9	Do you recommend regular bloodwork for this patient?	1. Every 6 months<br>2. Every year<br>3. No regular bloodwork, only as needed<br><br>SINGLE CHOICE
D9a	**If D9=1 or 2:**<br>Are you planning to continue with this bloodwork screening?	1. Yes<br>2. No -> **if selected:** Why? ___________
Q1	Did this patient receive another treatment for OA pain before starting Librela?	**1.** Yes -> **Continue with P1**<br>**2.** No -> **Continue with T1**

**PREVIOUS TREATMENT (the OA Pain treatment before starting Librela)**		
P1	Patient previous treatment categories for OA pain:<br>MULTIPLE CHOICE	1. Oral treatment<br>2. Injectable treatment<br>3. Nutraceutical<br>4. Physical therapy<br>5. Other, please specify: _______
P2	Patient previous OA pain treatment details	• **If P1=1:**<br>• Oral treatment details:<br>• Type (NSAID/ Steroids etc.): ___________<br>• Brand: ______________<br>• Dose: ______ mg/ kg<br>• Treatment Start date: __/__/___ **(DD/MM/YYYY)**<br>• Treatment Stop date, if applicable: __/__/___ **(DD/MM/YYYY)**<br>•<br>• **If P1=2:**<br>• Injectable treatment details:<br>• Type (NSAID/ Cartrophen/ Steroids etc.): ___________<br>• Brand: ______________<br>• Dose: ______ mg/ kg<br>• Treatment Start date: __/__/___ **(DD/MM/YYYY)**<br>• Treatment Stop date, if applicable: __/__/___ **(DD/MM/YYYY)**<br>•<br>• **If P1=3:**<br>• Nutraceutical treatment details:<br>• Brand: ______________<br>• Dose: ______ mg/ kg

		• Treatment Start date: __/__/___ (DD/MM/YYYY)<br>• Treatment Stop date, if applicable: __/__/___ (DD/MM/YYYY)<br>•<br>• **If P1=5:**<br>• Other treatment details:<br>• Brand: _______________<br>• Dose: ______ mg/ kg<br>• Treatment Start date: __/__/___ (DD/MM/YYYY)<br>• Treatment Stop date, if applicable: __/__/___ (DD/MM/YYYY)<br>•

• **OA PAIN TREATMENTS SINCE STARTING PATIENT ON LIBRELA**		
T1	What is the dosing frequency of Librela you recall that you recommended for this patient?	1. Monthly<br>2. Other, please specify frequency: ______
T2	Librela treatment details for this patient.<br><br>PLEASE CHECK THE PATIENT'S FILE AND DETAIL ALL ADMINISTRATIONS WITH LIBRELA	•<br>• **FIRST ADMINISTRATION OF LIBRELA**<br>• T2a. Total 1mL Vial/s Delivered: _______<br>•<br>• **ALLOW MULTIPLE ENTRIES FROM BELOW LIST, Allow numeric entries for the following between 0-1 (i.e. 5mL, 1mL,.75mL):**<br>• T2a. Form:<br>• 1. 5mg, ____ _mL Delivered (1mL Vial)<br>• 2. 10mg, _____mL Delivered (1mL Vial)<br>• 3. 15mg,____ _mL Delivered (1mL Vial)<br>• 4. 20mg,___ __mL Delivered (1mL Vial)<br>• 5. 30mg, ____ mL Delivered (1mL Vial)<br>•<br>• T2b. Treatment date: __/__/___ (DD/MM/YYYY)
		• **SECOND ADMINISTRATION OF LIBRELA (REPEAT QUESTIONS T2a-T2b)**<br>•<br>• **REPEAT QUESTIONS T2a-T2b FOR AS MANY ADMINISTRATIONS AS NEEDED**

T3	What were the reasons for starting treatment with Librela in this patient?	1. Decrease the number of medications for this patient<br>2. Did not achieve complete pain reduction/ efficacy with previous treatment<br>3. Safety concerns with previous treatments<br>4. Diagnostic blood test results showing negative impact on organ function<br>5. Patient starting therapy/ no previous treatment<br>6. Improve compliance<br>7. Librela became available<br>8. Pet owner request<br>9. Others, please specify: ____________<br><br>**MULTIPLE CHOICES. ROTATE OPTIONS**
T4	When is the patient scheduled to receive their next injection?	Date: __/__/___ **(DD/MM/YYYY)**
T5	If the patient did not receive the recommended monthly dosing, why was this?	1. Cost<br>2. Scheduling difficulty/busy lifestyle<br>3. Dog didn't seem to need it yet<br>4. Pet owner chose to discontinue Librela<br>5. Not applicable, all monthly doses received (**EXCLUSIVE OPTION**)<br>6. I recommend less frequent dosing<br>7. Other, please specify: ________<br>•<br><br>**MULTIPLE CHOICE**<br><br>•
T6	How satisfied are you with the results following the treatment with Librela?	• 1=Not satisfied at all<br>• 2<br>• 3<br>• 4<br>• 5<br>• 6<br>• 7<br>• 8<br>• 9<br>• 10=Fully satisfied
T7	**If T6=1,2,3 or 4**: Why are you not satisfied with the results in this patient? **Open-ended question**	• ____________________________
T8	Since this patient started on Librela, what other medications is	1. I added these product (s) to the treatment, please detail the change and the reason:<br><br>Product 1 ________ Reason: _________

	this patient receiving as part of their osteoarthritis management?	Product 2 ________ Reason: _________<br><br>**Add as many input boxes as needed**<br><br>2. I continued with:<br>Product 1 ________ for ____ days<br>Product 2 ________ for ____ days<br><br>**Add as many input boxes as needed**<br><br>3. I discontinued with:<br>Product 1 ________ for __ weeks Reason:_______<br>Product 2 ________ for __ weeks Reason:_______<br><br>**Add as many input boxes as needed**<br><br>4. No other treatment since starting Librela (EXCLUSIVE OPTION)<br>•<br><br>MULTIPLE CHOICE

➔ REPEAT D1-T8 FOR 5-7 PATIENTS

T9. What is the cost that you charge a dog owner for one Librela dose for a 20kg dog? Please provide all that is applicable.

**Visit Fee**	_____ (local currency)
**Injection Fee**	_____ (local currency)
**Librela Cost**	_____ (local currency)
**VAT or other taxes**	_____ (local currency)

End of form.
